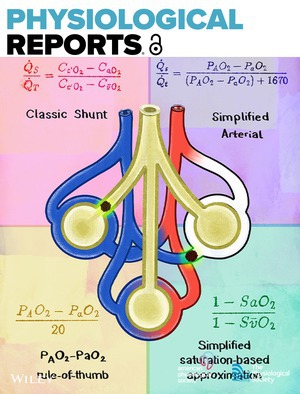# Cover Image

**DOI:** 10.14814/phy2.70806

**Published:** 2026-03-02

**Authors:** John G. Toffaletti, Gerald S. Zavorsky

## Abstract

The cover image is based on the article *Calculating pulmonary shunt fraction using standard clinical measurements* by Gerald Zavorsky et al., https://doi.org/10.14814/phy2.70763